# Ploidy and Hybridity Effects on Growth Vigor and Gene Expression in *Arabidopsis thaliana* Hybrids and Their Parents

**DOI:** 10.1534/g3.112.002162

**Published:** 2012-04-01

**Authors:** Marisa Miller, Changqing Zhang, Z. Jeffrey Chen

**Affiliations:** Section of Molecular Cell and Developmental Biology, Center for Computational Biology and Bioinformatics, and Institute for Cellular and Molecular Biology, University of Texas at Austin, Austin, Texas 78712

**Keywords:** polyploidy, heterosis, gene expression, hybrids, circadian clock

## Abstract

Both ploidy and hybridity affect cell size and growth vigor in plants and animals, but the relative effects of genome dosage and hybridization on biomass, fitness, and gene expression changes have not been systematically examined. Here we performed the first comparative analysis of seed, cell, and flower sizes, starch and chlorophyll content, biomass, and gene expression changes in diploid, triploid, and tetraploid hybrids and their respective parents in three *Arabidopsis thaliana* ecotypes: Columbia, C24, and Landsberg *erecta* (L*er*). Ploidy affects many morphological and fitness traits, including stomatal size, flower size, and seed weight, whereas hybridization between the ecotypes leads to altered expression of central circadian clock genes and increased starch and chlorophyll content, biomass, and seed weight. However, varying ploidy levels has subtle effects on biomass, circadian clock gene expression, and chlorophyll and starch content. Interestingly, biomass, starch content, and seed weight are significantly different between the reciprocal hybrids at all ploidy levels tested, with the lowest and highest levels found in the reciprocal triploid hybrids, suggesting parent-of-origin effects on biomass, starch content, and seed weight. These findings provide new insights into molecular events of polyploidy and heterosis, as well as complex agronomic traits that are important to biomass and seed production in hybrid and polyploid crops.

Hybridization within and between species is a naturally occurring process and is estimated to take place in at least 25% of plant species (Chen 2010; Mallet 2004). A common consequence of hybridization is hybrid vigor, or heterosis, which refers to superior performance in biomass, yield, or other agronomic traits in hybrids relative to one or both parents (Birchler *et al.* 2003). Hybrid vigor is of great importance to crop production as 65% or more crops are grown as hybrids, and the yield increase in hybrids ranges from 15 to 50% (Lippman and Zamir 2007). Although hybrid vigor has been known since the time of Charles Darwin (Darwin 1876), the molecular basis remains to be elucidated (Birchler *et al.* 2010; Chen 2010). It is predicted that changes in gene expression and regulatory networks are associated with heterotic effects in hybrids (Birchler *et al.* 2003). Indeed, many genes are expressed differently from the mid-parent value (MPV) in the hybrids of maize (Auger *et al.* 2005; Guo *et al.* 2004; Springer and Stupar 2007; Swanson-Wagner *et al.* 2006), rice (He *et al.* 2010), and *A. thaliana* (Groszmann *et al.* 2011), some of which are associated with changes in DNA methylation and small RNAs (Groszmann *et al.* 2011; He *et al.* 2010).

Hybrids are genetically unstable, making it difficult to dissect the molecular basis for heterosis. Allotetraploids, in which the chromosomes in interspecific hybrids are doubled, are genetically stable. As a result, the heterozygosity and hybrid vigor are permanently fixed in allopolyploids (Chen 2010). Genome-wide nonadditive gene expression has been documented in *Arabidopsis* allotetraploids that are derived from *Arabidopsis thaliana* and *Arabidopsis arenosa* (Wang *et al.* 2004, 2006). Similar gene expression changes have also been found in other allopolyploids of *Brassica* (Marmagne *et al.* 2010), cotton (Adams *et al.* 2003; Flagel *et al.* 2008), *Senecio* (Hegarty *et al.* 2006), *Spartina* (Chelaifa *et al.* 2010), *Tragopogon* (Buggs *et al.* 2009; Tate *et al.* 2006), and wheat (Chague *et al.* 2010; Pumphrey *et al.* 2009). A recent study found a link between altered circadian rhythms and increased growth vigor in *Arabidopsis* allotetraploids and *A. thaliana* F1 hybrids (Ni *et al.* 2009). Specifically, epigenetic regulation of clock genes in the allotetraploids is directly linked to increased expression of downstream genes and metabolic pathways, leading to increased levels of chlorophylls, starch, and sugars during vegetative growth. In plants and animals, circadian clock regulation plays a central role in optimizing metabolic pathways and increasing fitness (Dodd *et al.* 2005; Wijnen and Young 2006). In plants, many pathways, including chlorophyll biosynthesis and starch metabolism, are controlled by clock regulation (Harmer *et al.* 2000; Smith *et al.* 2004). Starch degradation at night is also diurnally regulated and related to plant growth (Graf *et al.* 2010). But starch accumulation during the day and degradation at night are not mutually exclusive, as has been deliberately argued (Graf and Smith 2011). The more starch accumulates during the day, the more starch can be degraded at night, and plants can grow more rapidly and accumulate more biomass (Chen 2010).

Growth vigor in allopolyploids is confounded by the effects of increased levels of ploidy and genomic hybridity. There is no obvious growth vigor in *A. thaliana* autotetraploids, although genome dosage is increased relative to diploids (Chen 2010). In maize, the number of nonadditively expressed genes and the degree of their expression increase from duplex to quadruplex hybrids (Riddle *et al.* 2010). These gene expression trends are consistent with observed phenotypic variation, but the mechanistic connections remain unknown. To test the relative impact of ploidy and hybridity on heterosis, we generated a series of reciprocal diploid, triploid, and tetraploid hybrids using *A. thaliana* ecotypes (Col, C24, and L*er*) of diploid and isogenic tetraploid parents. We first validated these genetic materials using chromosome counts, flow cytometry, and DNA markers. We then evaluated biomass, stomatal size, flower morphology, and seed size and weight in these lines. Finally, we tested whether expression of circadian clock genes is correlated with starch and chlorophyll content and biomass in these hybrids. The results provide novel insights into the effects of genomic hybridization, ploidy, and circadian gene expression on biomass, cell and seed size, and starch metabolism in *Arabidopsis* intraspecific hybrids. The information is of practical use for the improvement of biomass and seed production in vegetable and food crops.

## Materials and Methods

### Plant growth and materials

Two sets of reciprocal hybrids were generated in each genotypic combination using *A. thaliana* ecotypes Columbia (Col), C24, and Landsberg *erecta* (L*er*) diploid (2X) and isogenic autotetraploids (4X) as parents (Col2, Col4, L*er*2, L*er*4, C24-2, and C24-4). These hybrids include (1) reciprocal diploid hybrids [Col2XC24-2 and C24-2XCol2 (by convention the maternal parent is listed prior to the paternal parent in a genetic cross) and Col2XL*er*2 and L*er*2XCol2], (2) reciprocal triploid hybrids (Col2XC24-4 and C24-4XCol2; Col4XC24-2 and C24-2XCol4; and Col2XL*er*4), and (3) reciprocal tetraploid hybrids (Col4XC24-4 and C24-4XCol4; Col4XL*er*4 and L*er*4XCol4). Crossing within (parents) and between *A. thaliana* ecotypes was carried out by removing immature anthers from unopened flower buds in the maternal ecotype and fertilizing the stigma with pollen from freshly opened flowers of the paternal ecotype. Plants were grown on soil in 16/8-h (light/dark) cycles (Wang *et al.* 2004).

### Chromosome spreads and flow cytometry

Chromosome spreads of young flower buds were prepared as previously described (Lysak *et al.* 2006) and stained with 4′,6-diamidino-2-phenylindole (DAPI). Slides were observed using a light microscope (Zeiss Axiovert 200 M).

For flow cytometry samples, 70 mg leaves from seedlings were collected and processed as previously described (Galbraith *et al.* 1983), except that the samples were filtered through 30 μm Partec CellTrics filters and were stained using propidium iodide (PI) at a concentration of 100 μg/ml. Samples were stained for 40 to 60 min before performing flow cytometry. Flow cytometry was performed on a BD Biosciences FACSCalibur flow cytometer (BD Biosciences, San Jose, California), and data were acquired using CellQuestPro software (Becton, Dickinson and Company, Franklin Lakes, New Jersey). Samples were run on low pressure long enough to acquire clear peaks (5–10 min). Because PI (emission maximum 639 nm) was used as the DNA stain, the FL2 detector (564–606 nm) was used to measure fluorescence. The fluorescence 2-area (FL2-A), a measurement of integrated fluorescence signal, was used as the parameter linearly correlated to DNA content (Dart *et al.* 2004). *A. thaliana* diploid leaf tissues were used to calibrate the instrument prior to running the samples. Noise signals derived from subcellular debris were eliminated by gating (Ng *et al.* 2011).

### DNA and RNA extraction and analysis

Total genomic DNA was isolated from leaves by grinding in extraction buffer (0.2 M Tris-HCl, pH 7.5; 0.25 M NaCl; 25 mM EDTA; and 0.5% SDS). The supernatant was then precipitated with isopropanol, and the pellet was rinsed with 75% ethanol. Following centrifugation, the pellet was resuspended in sterile water. PCR was used to amplify DNA fragments with length polymorphisms between different ecotypes and in the hybrids. The marker used to distinguish ecotypes, nga106, is described in Bell and Ecker (1994). PCR primers were obtained in TAIR. Approximately 500 ng genomic DNA was used for PCR using AmpliTaq DNA Polymerase (Applied Biosystems, Foster City, California).

Tissue collected for gene expression analysis was collected from plants before bolting (6-8 rosette leaves) at indicated Zeitgeber time (ZT0 = dawn) (McClung 2006). Total RNA was extracted using Concert Plant RNA Reagent (Invitrogen, Carlsbad, California) according to the manufacturer's instructions. Total RNA was digested with RQ1 RNase-Free DNase (Promega, Madison, Wisconsin) to remove any DNA. cDNA was synthesized from the DNase-treated RNA using Superscript III (Invitrogen). One microliter of diluted cDNA was used for quantitative RT-PCR analysis using the primer pairs listed in Table S1 in an ABI7500 machine (Applied Biosystems) as described in Chen *et al.* (2004), except that *ACT* was used as an internal control to calculate the relative expression levels in three biological replications.

### Biomass measurement

Whole rosettes from hybrids and parents were harvested at approximately three weeks of age (before bolting) and placed in Lawson #217 hybridization bags (Lawson Bags, Northfield, Illinois). Dry weights from rosette leaves were determined after drying the plants at 80° for 24 h. Each rosette in three biological pools was weighed individually, and the average was used to calculate standard deviations.

### Stomatal measurement

Stomatal imprints were taken from the abaxial side of leaves from plants before bolting. Young leaves were placed onto a small drop of cyanoacrylate adhesive (“Superglue”) and gentle pressure was applied for approximately 30 sec. Leaves were then gently removed, and the imprint was allowed to dry for at least 10 min before viewing with a Leica DM LB microscope. Stomatal size was determined by measuring 20 stomata per genotype lengthwise. Stomatal density was defined as the number of stomata per square millimeter.

### Seed size and weight analysis

Average seed weight was determined by weighing mature dry seeds in batches of 150. The weights of three batches were measured for each seed lot using an analytical balance. Sizes of parent and hybrid seeds were analyzed by separating batches of 150 seeds using a series of fine wire sieves. Sieve mesh sizes 40, 45, 50, 60, 70, and 80 (Fisher Scientific, Waltham, Massachusetts) with exclusion sizes of 425, 355, 300, 250, 212, and 180 μm, respectively, were used for each analysis. Seeds retained by each sieve were counted and a weighted average was calculated. Three batches of seeds were measured for each genotype.

### Starch and chlorophyll analysis

Starch and chlorophyll extraction and analyses were performed as in Ni *et al.* (2009), except that 300 mg tissue was used per biological replication.

## Results

### Validation of chromosomal content and ploidy levels of F1 hybrids

**Figure 1:**
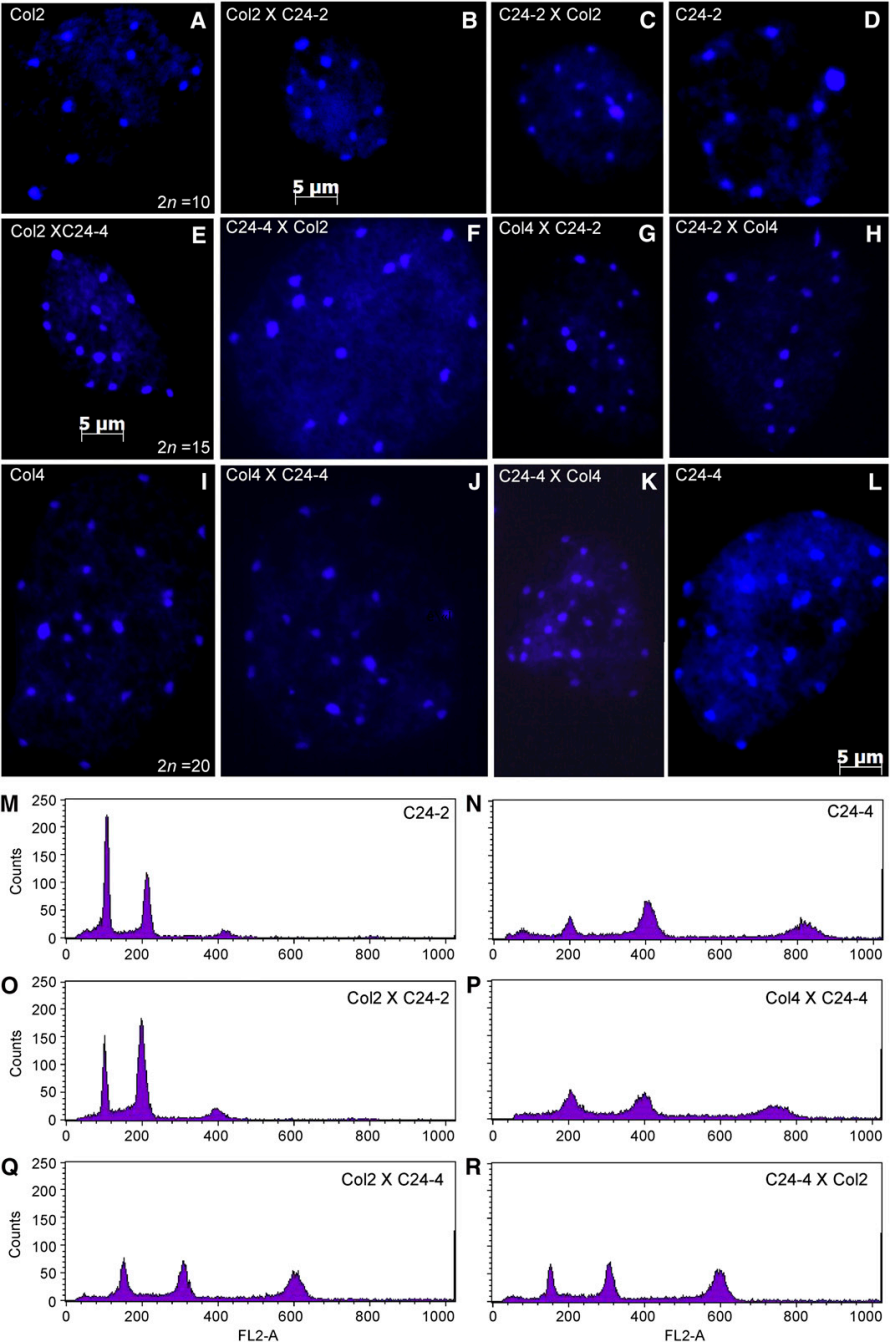
Validation of ploidy and genotype in *Arabidopsis thaliana* hybrids and their parents (Col and C24). (A–L) Chromosome spreads were prepared from ploidy hybrids and their parents and stained with DAPI. (A) Col2, (B) Col2XC24-2, (C) C24-2XCol2, (D) C24-2, (E) Col2XC24-4, (F) C24-4XCol2, (G) Col4XC24-2, (H) C24-2XCol4, (I) Col4, (J) Col4XC24-4, (K) C24-4XCol4, and (L) C24-4. One representative nucleus per genotype is shown (scale bar = 5 μm). (M–R) Flow cytometry analysis of nuclei from leaves of hybrids and parents. (M) C24-2, (N) C24-4, (O) Col2XC24-2, (P) Col4XC24-4, (Q) Col2XC24-4, and (R) C24-4XCol2. Filtered nuclei were stained with propidium iodide and analyzed using flow cytometry (X-axis = fluorescence intensity, Y axis = nuclei counts).

Three different methods were employed to ensure that the F1 hybrids used for this study (see *Materials and Methods*) were true hybrids with the expected ploidy levels (Figure 1, A–L). To determine the ploidy level of the hybrids, chromosome spreads were prepared from young flower buds and stained with DAPI. All nuclei observed had the expected number of chromosomes. For example, all triploid hybrids contained 15 chromosomes (Figure 1, E–H), and all tetraploids and tetraploid hybrids had 20 chromosomes (Figure 1, I–L). Flow cytometry was also used to examine the genome composition (Figure 1, M–R and supporting information, Figure S1, A–F). Consistent with the previous finding (Galbraith *et al.* 1991), endoreduplication is commonly observed in leaves of *A. thaliana* diploids (Figure 1M and Figure S1A). The level of endoploidy was proportionally increased from diploids to tetraploids (Figure 1N and Figure S1B), consistent with data in a recent study (Ng *et al.* 2011). In addition, no significant difference in the level of endoreduplication was observed in the comparison between hybrids and parents at the same ploidy levels (*e.g.* diploid parents and hybrids; Figure 1, M and O). As expected, the endoploidy level in the triploid hybrids was between those in the diploids and tetraploids (Figure 1, Q and R, and Figure S1, E and F). Genomic changes in different ploidy levels of *A. thaliana* are proportional to their endoploidy levels, suggesting that molecular changes in *A. thaliana* polyploids are not compromised by endoreduplication. However, neither chromosome counts nor flow cytometry in hybrids can rule out a possibility of selfing. Thus, the genomic content of diploid and tetraploid hybrids was further validated using simple sequence length polymorphism (SSLP) between Col and C24 or Col and L*er* ecotypes (Figure S1, G and H). All hybrids genotyped showed presence of both parental fragments.

### Effects of genome dosage and hybridization on biomass and cell size in F1 hybrids at different ploidy levels

**Figure 2:**
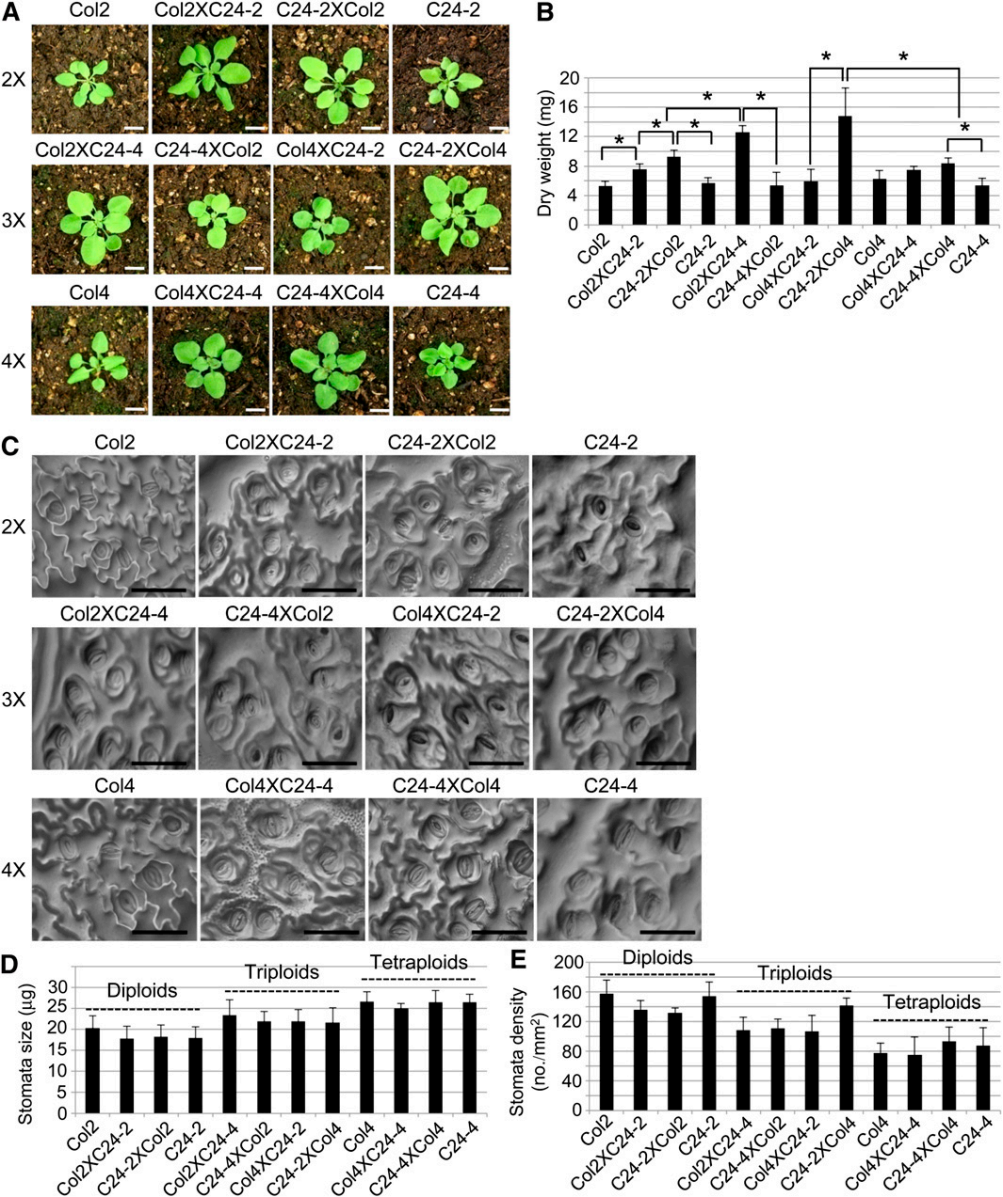
Relative effects of genome dosage and hybridization on rosette size, dry weight, stomata size, and stomata density on ploidy hybrids and their parents (Col and C24). (A) Morphological vigor in diploid, triploid, and tetraploid hybrids and their parents. Images are of 15-day-old plants (bar = 1 cm). (B) Aerial biomass in diploids, triploid, and tetraploid hybrids and parents (n = 4–5 plants). Asterisks indicate statistical significance (*P* = 0.05). (C) Stomatal imprints of diploid, triploid, and tetraploid hybrids and their parents (bar = 50 μm) (D) Stomatal size (n = 20). (E) Stomatal density per mm^2^ (n = 150–200) in diploid, triploid, and tetraploid hybrids and their parents. Error bar ± SD.

Given that some hybrids formed between *A. thaliana* ecotypes do not display obvious growth vigor, reciprocal hybrids of different ploidy levels were generated between Col and C24 or Col and L*er* because the diploid hybrids are shown to display growth vigor (Barth *et al.* 2003; Meyer *et al.* 2004). The diploid hybrids (ColXC24 and ColXL*er)* both displayed increased levels of biomass when compared with their parents (Figure 2, A and B, and Figure S2). These hybrids were approximately 50–100% larger than their respective parents. The biomass between reciprocal hybrids was also significantly different but to a lesser degree (Figure 2B). Interestingly, the triploid hybrids showed a much greater size disparity between reciprocal crosses (Figure 2, A and B). In particular, both triploid hybrids with a tetraploid father are 1–1.5 times larger than the triploid hybrids with a tetraploid mother. This obvious parent-of-origin effect on organismal growth has also been shown in triploid humans (McFadden *et al.* 1993). The size difference between reciprocal triploid hybrids became less dramatic after flowering. The triploid hybrids with a tetraploid mother had similar biomass relative to the diploid and tetraploid parents. A similar phenomenon was also observed in Col2XL*er*4 triploid hybrids that displayed higher biomass than the parents (Figure S2). Somewhat unexpectedly, tetraploid hybrids were slightly larger than the tetraploid diploids but not significantly larger than the diploid hybrids. In the reciprocal tetraploid hybrids, the biomass was slightly different (Figure 2, A and B).

**Figure 3:**
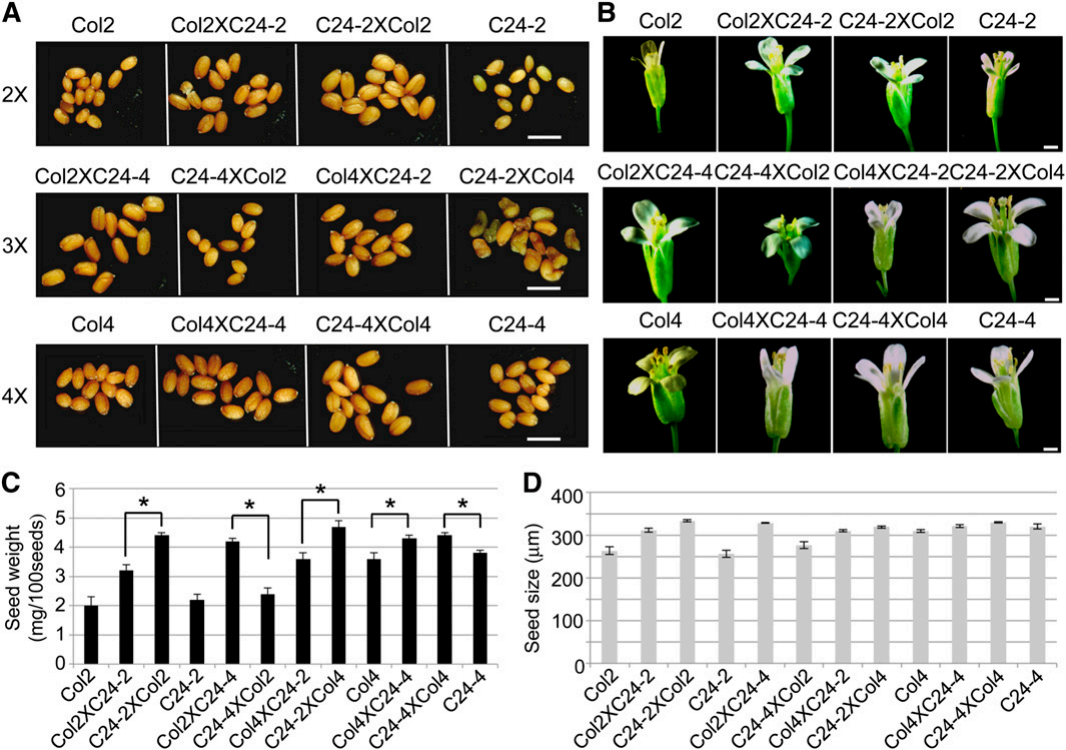
Seed size, weight, and flower size of ploidy hybrids and their parents. (A) Seeds of diploid, triploid, and tetraploid hybrids and their parents (bar = 1 mm). (B) Flower size in diploid, triploid, and tetraploid hybrids and their parents (bar = 1 mm). (C) Seed weight per 100 seeds (n = 150). Asterisks indicate statistical significance (*P* = 0.05). (D) Seed size measured using mesh sieves (n = 150). Error bar ± SD.

Increased biomass is probably due to increased cell size, number, or both. To test this, we measured the stomatal size and density in these lines. Stomata are epidermal structures that consist of two guard cells surrounding a pore that allows for gas exchange (Nadeau 2009). To determine the effects of hybridization and genome dosage on stomatal size and density, stomatal imprints were taken of diploid, triploid, and tetraploid hybrids between Col and C24 ecotypes and quantified (Figure 2, C–E). The stomatal size increased from diploids, to triploids, to tetraploids, whereas the density was negatively correlated with the genome dosage. No obvious difference was found between hybrids and parents at different ploidy levels, nor was there a difference between the reciprocal hybrids, with a couple of exceptions. The triploid hybrid (C24-2XCol4) showed higher stomatal density than other triploid hybrids. The C24-2XCol4 triploid seeds are viviparous and somewhat green (Figure 3A). This is likely caused by growing of embryo or underdevelopment of seed coat. Both Col and C24 diploids had higher cell density than their hybrids, suggesting larger cells in hybrids than in the parents.

Although ploidy did not obviously affect biomass, seed size and weight, as well as flower size, are more strongly affected by the ploidy levels. In general, increasing the ploidy level is positively correlated with increasing the flower size and seed weight, so tetraploids had heavier seeds and larger flowers than diploids (Figure 3, A–D). The difference in seed size in some comparisons was not dramatic probably because of a crude measurement method. Interestingly, seeds in one diploid hybrid (C24-2XCol2) were nearly as large as tetraploid hybrid seeds and 20–40% larger and 60–120% heavier than their respective parents (Figure 3, C and D). There was a significant parent-of-origin effect on seed weight in reciprocal diploid hybrids, although this effect on seeds was not obvious in the reciprocal tetraploid hybrids. The parent-of-origin effect was most obvious in triploid hybrid seeds. Similar to vegetative growth and biomass, the triploid hybrids with a tetraploid father produced larger and heavier seeds than the triploid hybrids with a tetraploid mother. Flower size in triploid hybrids also showed this same trend. This obvious parent-of-origin effect is interesting and remains to be tested.

### Effects of genome dosage and hybridization on expression of circadian clock genes and starch metabolic genes and output traits in hybrids

**Figure 4:**
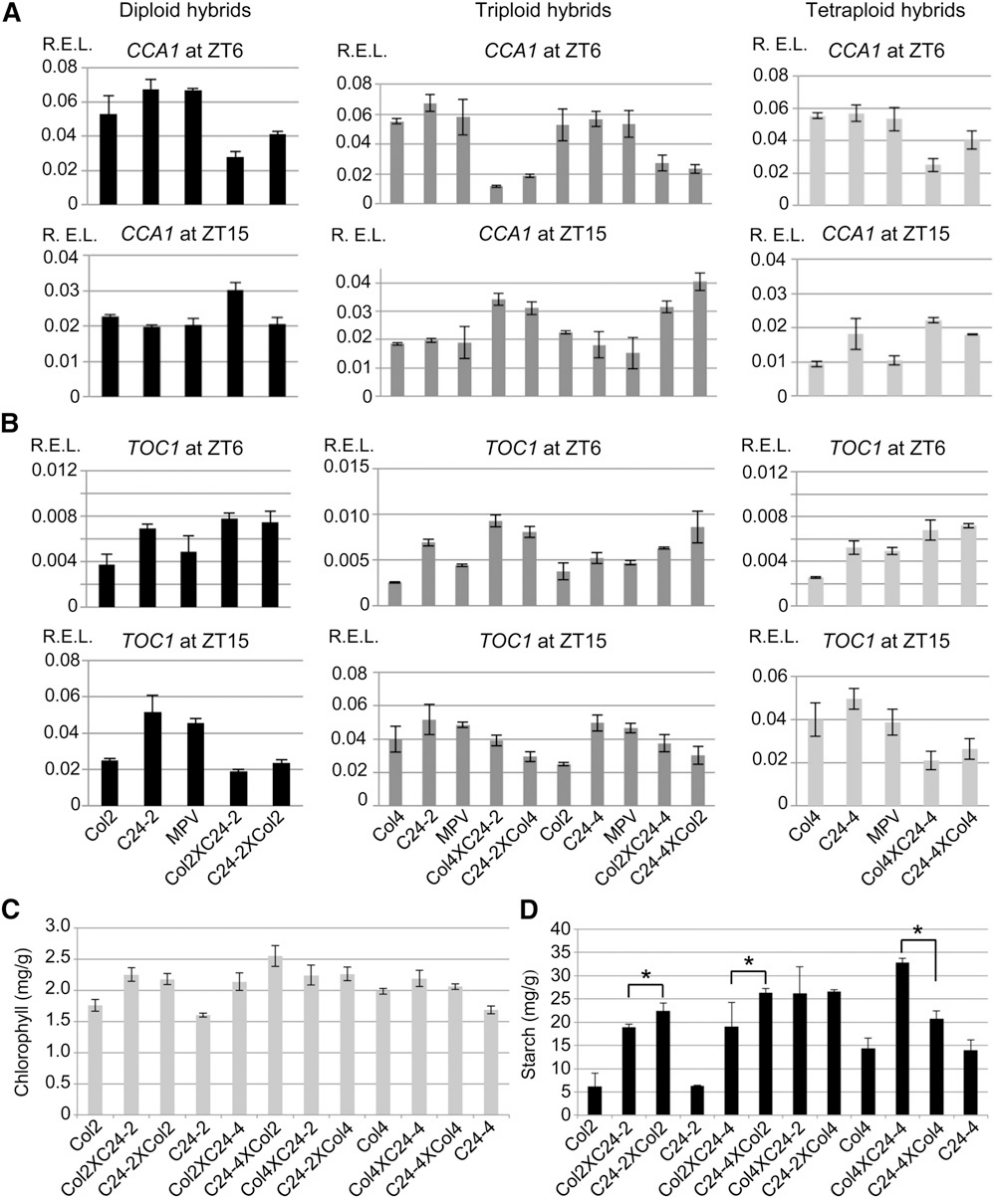
Expression of the circadian clock genes *CCA1* and *TOC1* at ZT6 and ZT15, and starch and chlorophyll content in ploidy hybrids and their parents. Quantitative RT-PCR analysis of (A) *CCA1* and (B) *TOC1* (n = 3, *ACT* as an internal control) in diploid, triploid, and tetraploid hybrids and their parents. Error bar ± SD. The (C) chlorophyll content (n = 2) and (D) starch content (n = 3) in diploid, triploid, and tetraploid hybrids and their parents. Asterisks indicate statistical significance (*P* = 0.05). Error bar ± SD.

A recent study showed that in *Arabidopsis* hybrids and allopolyploids epigenetic regulation of core clock genes is directly linked to increased expression of downstream genes and metabolic pathways, leading to increased amounts of chlorophylls, starch, and sugars during vegetative growth (Ni *et al.* 2009). Expression changes in circadian clock genes and their regulatory networks are also related with yield quantitative trait loci (QTL) in super-hybrid rice (Song *et al.* 2010). In *Arabidopsis*, the core loop of the circadian clock is composed of the transcription factors Circadian Clock Associated 1 (CCA1) and Late Elongated Hypocotyl (LHY), which are positively regulated by Timing of Cab Expression 1 (TOC1) and CCA1 Hiking Expedition (CHE) (McClung 2006, 2010; Pruneda-Paz and Kay 2010). CCA1 and LHY are negative regulators of *TOC1*, and TOC1 and CHE positively regulate the expression of *CCA1* and *LHY*. To determine the effects of ploidy and hybridization on gene expression and circadian-mediated growth vigor, we examined expression of core clock regulators and their downstream genes in hybrids with different ploidy levels. Consistent with the published data (Ni *et al.* 2009), in diploid hybrids between Col and C24 *CCA1*, transcript levels were reduced 1.5- to 2-fold relative to the MPV at ZT6 and slightly upregulated at ZT15 (Figure 4A). Conversely, *TOC1* was upregulated at ZT6 and downregulated at ZT15 relative to MPV (∼2.5 fold) (Figure 4B). We further found a similar trend of CCA1 repression and TOC1 upregulation in the triploid and tetraploid hybrids. *CCA1* was repressed 2- to 4-fold in the triploid hybrids and ∼1.5- to 2-fold in the tetraploid hybrids at ZT6 and upregulated at ZT15. *TOC1* was upregulated at ZT6 and downregulated at ZT15 in the triploid hybrids. Interestingly, the levels of *CCA1* repression and *TOC1* upregulation were different between reciprocal hybrids in the diploid, triploid, and tetraploid levels, respectively, which is generally correlated with parent-of-origin effects on biomass (Figure 2, A and B). However, some changes were not obvious between ploidy levels. The molecular connection between changes in clock genes and biomass in reciprocal crosses needs to be further investigated.

Consistent with similar rosette size, parental lines of diploid and tetraploid Col and C24 showed similar changes in the expression levels of *CCA1* and *TOC1* (Figure 4, A and B), which provides another piece of correlative data between circadian rhythms and growth vigor (Ni *et al.* 2009). Hybrids between Col and L*er* also showed a general trend of *CCA1* repression at ZT6 and upregulation at ZT15, but to a lesser extent (Figure S3). This is probably related to the level of hybrid vigor that is dependent on genotypic combinations (East 1936): the larger the genetic distance, the higher the level of heterosis, if hybrids can be formed (Chen 2010).

We also examined expression of downstream genes and circadian-mediated output traits, including starch and chlorophyll content, in diploid, triploid, and tetraploid hybrids. *PORA* and *PORB* catalyze the only known light-requiring step of tetrapyrrole biosynthesis (Reinbothe *et al.* 1996). Both *PORA* and *PORB* contain evening elements (EE) and CCA1 binding sites (CBS) within their promoters, and they are the targets of CCA1 (Harmer *et al.* 2000). Both *PORA* and *PORB* were upregulated in the diploid and triploid hybrids between Col and C24 (Figure S4) and between Col and L*er* (Figure S5), but they were less induced in the tetraploid hybrids than in the diploid and triploid hybrids. As a result, total chlorophyll content was increased in all hybrids relative to their respective parents, with a smaller level of increase in the tetraploid hybrids (Figure 4C and Figure S6).

In *Arabidopsis* allopolyploids, many EE- and CBS-containing genes involved in starch metabolism are also upregulated (Ni *et al.* 2009). We found that *DPE1*, *AMY3*, and *GWD3* were upregulated in the hybrids between Col and C24 and Col and L*er* (Figure S4 and Figure S5). However, the expression difference of starch metabolic genes was not obviously correlated with different ploidy hybrids. In general, starch content was increased in the diploid, triploid, and tetraploid hybrids, with the highest starch content in the tetraploid hybrid (Col4XC24-4) (Figure 4D). The starch content was significantly different in the reciprocal hybrids at diploid, triploid, and tetraploid levels, with one exception. Similar starch content was observed in Col4XC24-2 and C24-2XCol4 triploid hybrids, although the biomass and seed size were dramatically different between these crosses (Figures 2 and 3).

## Discussion

Our analysis of phenotypic variation and dosage regulation in some *Arabidopsis* intraspecific hybrids at diploid, triploid, and tetraploid levels has shown several interesting findings.

First, all hybrids tested, except for the triploid hybrids with a tetraploid mother, display biomass vigor compared to their respective parents. The highest biomass was found in the triploid hybrids with a diploid mother and a tetraploid father. This maternal effect is dependent on genotypes (*e.g.* C24 *vs.* Col). There appears to be a dosage compensation mechanism for ploidy effects on plant size. In maize, the plant size increases from haploid to triploid, but it decreases in the tetraploid (Riddle *et al.* 2006), which is consistent with smaller *A. thaliana* plants in the haploids than in the diploids (Henry *et al.* 2010; Ravi and Chan 2010).

Second, polyploids have bigger cells with relatively low density, which supports the previous notion of estimating polyploid plant frequency using stomatal size in fossil samples (Masterson 1994). Stomatal size is positively correlated with ploidy levels, independent of hybridization effects, except in the diploid hybrids, whereas stomatal density is negatively correlated with genome dosage, with a few exceptions probably because of genotypic effects. The data are consistent with that in *A. thaliana* and *A. lyrata* diploids and tetraploids (Beaulieu *et al.* 2009). No significant difference in stomatal cell size in the hybrids might suggest that hybrid plants increase cell number probably as a result of rapid cell division. However, this notion needs to be confirmed with a precise measure of cell size using other methods such as cell sorting.

Third, seed and flower size do not compensate for increased levels of ploidy because tetraploid seeds are bigger and heavier than diploid and triploid seeds, as observed in other plants species (Bretagnolle *et al.* 1995). Seed size is also increased in the hybrids relative to the parents in the same ploidy level. Modest seed size increases are also observed in ColXC24 and CviXL*er* F1 hybrids (Alonso-Blanco *et al.* 1999; Meyer *et al.* 2004) and in tomato hybrids (Ashby 1937). However, the effects of hybridity on seed size and weight in tetraploids are not obvious. Tetraploids seem to produce seeds at the maximum size level. It would be interesting to examine seed size variation in increased ploidy levels, such as hexaploid and octoploid plants.

A typical seed contains a diploid embryo and a triploid endosperm as a result of double fertilization in angiosperms (Haig and Westoby 1989; Stebbins 1976). The proper seed development requires an endosperm balance number of 2m:1p (maternal:paternal) genomes (Haig and Westoby 1989; Scott *et al.* 1998). In *A. thaliana*, increasing the paternal genome ratio (2m:2p) in paternal-excess triploids between a diploid “mother” and a tetraploid “father” (2X4) produces larger seeds. In contrast, increasing the maternal genome ratio (4m:1p) in maternal-excess triploids between a tetraploid mother and a diploid father (4X2) leads to smaller seeds (Scott *et al.* 1998; Tiwari *et al.* 2010). This phenomenon is consistent with the parental conflict model that explains genomic imprinting in mammals (Moore and Haig 1991; Tilghman 1999). In contrast to imprinting in the embryo, the endosperm is not genetically transmissible to offspring. In seeds, triploid endosperm is a maternal tissue that provides the nutrient reserve for seedling growth, as the placenta does in mammals, except that the latter is a diploid tissue. It is notable that larger seeds in these triploid hybrids with a diploid mother and tetraploid father also lead to increased biomass during early stages of vegetative growth. The molecular basis for this link remains to be investigated. One of the triploid hybrids (C24XCol4) produces greenish and viviparous seeds, which is probably related to expression disruption in genes, such as *TTG2*, in the seed coat in a dosage- and genotype-dependent manner (Dilkes and Comai 2004).

Fourth, expression of circadian clock genes is altered by hybridization irrespective of the ploidy levels, and genome dosage has no obvious effects on the overall level of clock gene expression. There is an overall correlation of *CCA1* repression and *TOC1* upregulation with increased levels of downstream genes, starch, and chlorophyll in hybrids at all ploidy levels tested. The degree of clock gene expression changes is increased from intraspecific hybrids to interspecific hybrids or allotetraploids (Ni *et al.* 2009), which is consistent with higher biomass vigor and starch content in interspecific hybrids than in intraspecific hybrids. Compared with genome dosage, genetic distance and hybridization are critical in determining the level of heterosis. However, expression of some transgenes (Finn *et al.* 2011) and endogenous genes (Auger *et al.* 2005; Wang *et al.* 2006) is dependent on genome dosage. This suggests that ploidy effects on some genes may not be directly related to growth and developmental phenotypes. Expression of clock genes is probably compensated by changes in genome dosage. That the overall vigor in the intraspecific tetraploid hybrids is much lower than in the allotetraploids makes this particularly evident (Ni *et al.* 2009).

Finally, changes in clock gene expression and starch content are different in reciprocal crosses at the diploid and tetraploid levels. This parent-of-origin effect appears to depend on maternal parents. However, in the triploid hybrids, the effects on starch content in the reciprocal crosses are opposite to the effects on biomass and seed size. This suggests that the effects of genome dosage and hybridization on seeds and overall plant size are uncoupled from starch metabolic pathways. In the reciprocal hybrids, upregulation of clock-regulated downstream genes is not always obvious, and there is less upregulation of downstream genes in intraspecific F1 hybrids than in allotetraploids (Ni *et al.* 2009). This indicates that there may be additional mechanisms or downstream effects in the F1 hybrids than there are in the allopolyploids. For example, small RNAs are shown to be differentially regulated in *Arabidopsis* allopolyploids (Ha *et al.* 2009) and *A. thaliana* hybrids (Groszmann *et al.* 2011) relative to the parents, and DNA methylation and gene expression changes are related in rice intraspecific hybrids (He *et al.* 2010). Together, these data should offer new insights into a better understanding of the complexity of growth vigor in intraspecific and interspecific hybrids, as well as in allopolyploids, that are of direct relevance to plant evolution and crop production.

## Supplementary Material

Supporting Information
