# Supplementary material for: Ploidy and Hybridity Effects on Growth Vigor and Gene Expression in Arabidopsis thaliana Hybrids and Their Parents
Source: G3 (Bethesda). 2012 Apr 1;2(4):505–13. doi: 10.1534/g3.112.002162 (PMC3337479; doi:10.1534/g3.112.002162)
Supplement: Supporting Information [file supp_2_4_505__index.html]

Supporting Information 

# Ploidy and Hybridity Effects on Growth Vigor and Gene Expression in *Arabidopsis thaliana* Hybrids and Their Parents

## Supporting Information for Miller, Zhang, and Chen, 2012

**Files in this Data Supplement:**

- Supporting Information - Figures S1-S6 and Table S1 (PDF, 1.8 MB)
- Figure S1 - Validation of genotype and ploidy in ColxC24 and ColXL*er* hybrids and parents (PDF, 1.1 MB)
- Figure S2 - A) Morphological vigor in ColXL*er* ploidy hybrids and their parents. B)Aerial biomass in ColXL*er* ploidy hybrids and their parents (bars 1 cm) (PDF, 412 KB)
- Figure S3 - Expression of circadian clock genes *CCA1* and *TOC1* in ColXL*er* ploidy hybrids and their parents at ZT6 and ZT15 (PDF, 113 KB)
- Figure S4 - Expression of the genes involved in chlorophyll and starch metabolism in ColXL*er* ploidy hybrids and their parents at ZT6 (PDF, 131 KB)
- Figure S5 - Expression of genes involved in chlorophyll and starch metabolism in ColXL*er* ploidy hybrids and their parents at ZT6 (PDF, 114 KB)
- Figure S6 - Starch and chlorophyll content in ColXL*er* ploidy hybrids and their parents (PDF, 112 KB)
- Table S1 - Primer sequences of *CCA1*, *LHY*, *TOC1* and genes involved in photosynthesis and starch metabolism for quantitative RT-PCR (PDF, 53 KB)
