# Supplementary material for: Ploidy and Hybridity Effects on Growth Vigor and Gene Expression in Arabidopsis thaliana Hybrids and Their Parents
Source: G3 (Bethesda). 2012 Apr 1;2(4):505–13. doi: 10.1534/g3.112.002162 (PMC3337479; doi:10.1534/g3.112.002162)
Supplement: Supporting Information [file supp_2.4.505_002162SI.pdf]

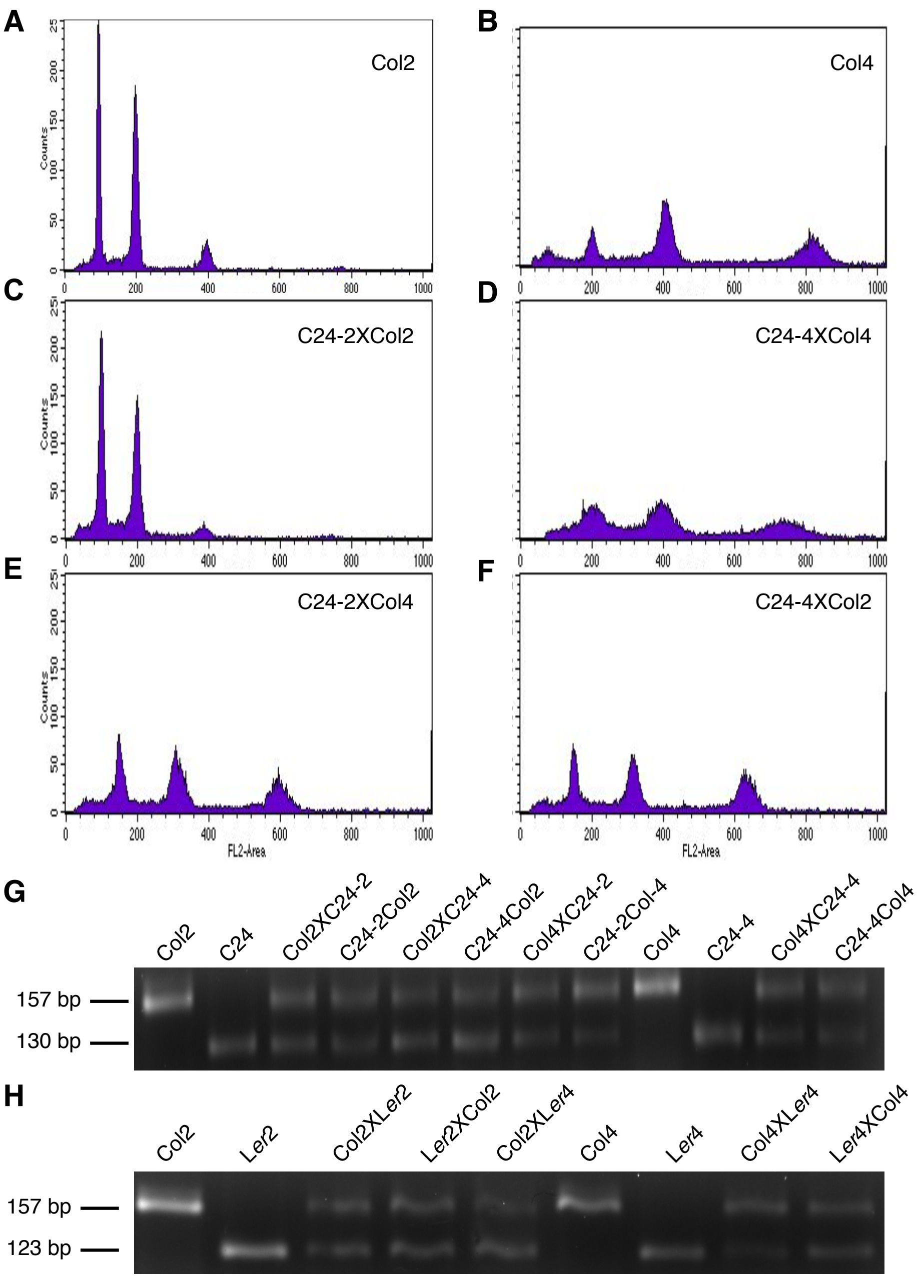

Figure S1 Validation of genotype and ploidy in ColxC24 and ColXL*er* hybrids and parents**.** (**A-F**) Flow cytometry analysis of nuclei from leaves of hybrids and parents. Filtered nuclei were stained with propidium iodide and analyzed using flow cytometry (X-axis = fluorescence intensity, Y axis = nuclei counts). (**G**) Genotyping in ColXC24 hybrids and parents using genomic DNA PCR. (**H**) Genotyping in ColXL*er* hybrids and parents using genomic DNA PCR. PCR length polymorphisms between the two different ecotypes were resolved on a 4% agarose gel to distinguish the two different genomes present in the hybrids.

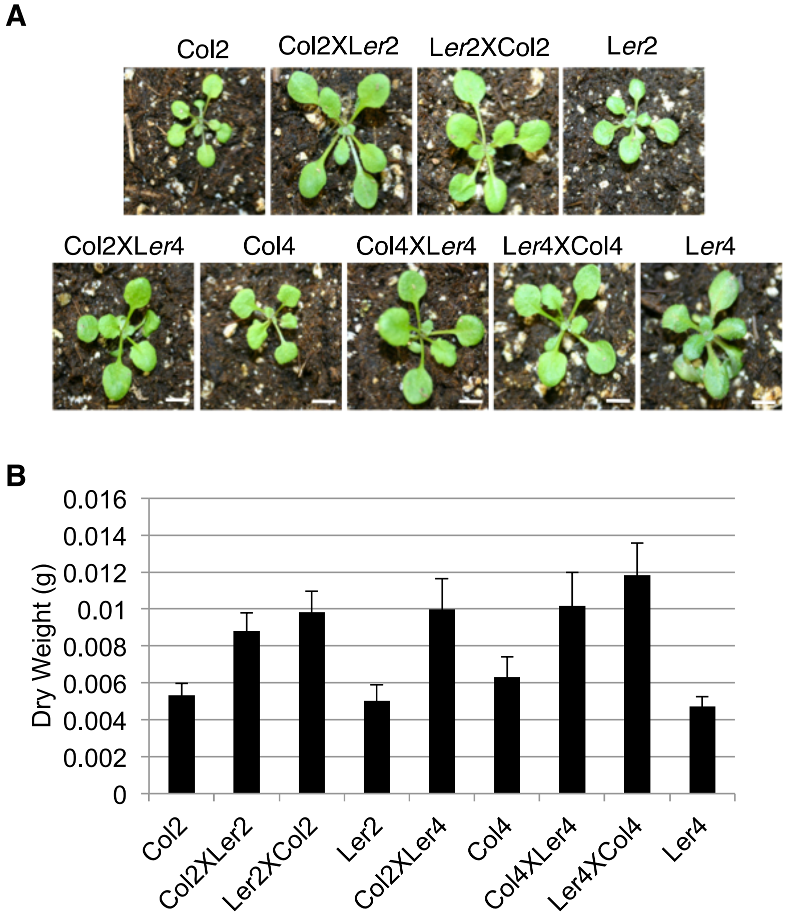

Figure S2 (**A**) Morphological vigor in ColXL*er* ploidy hybrids and their parents (bars 1 cm). (**B**) Aerial biomass in ColXL*er* ploidy hybrids and their parents. Error bars ± SD.

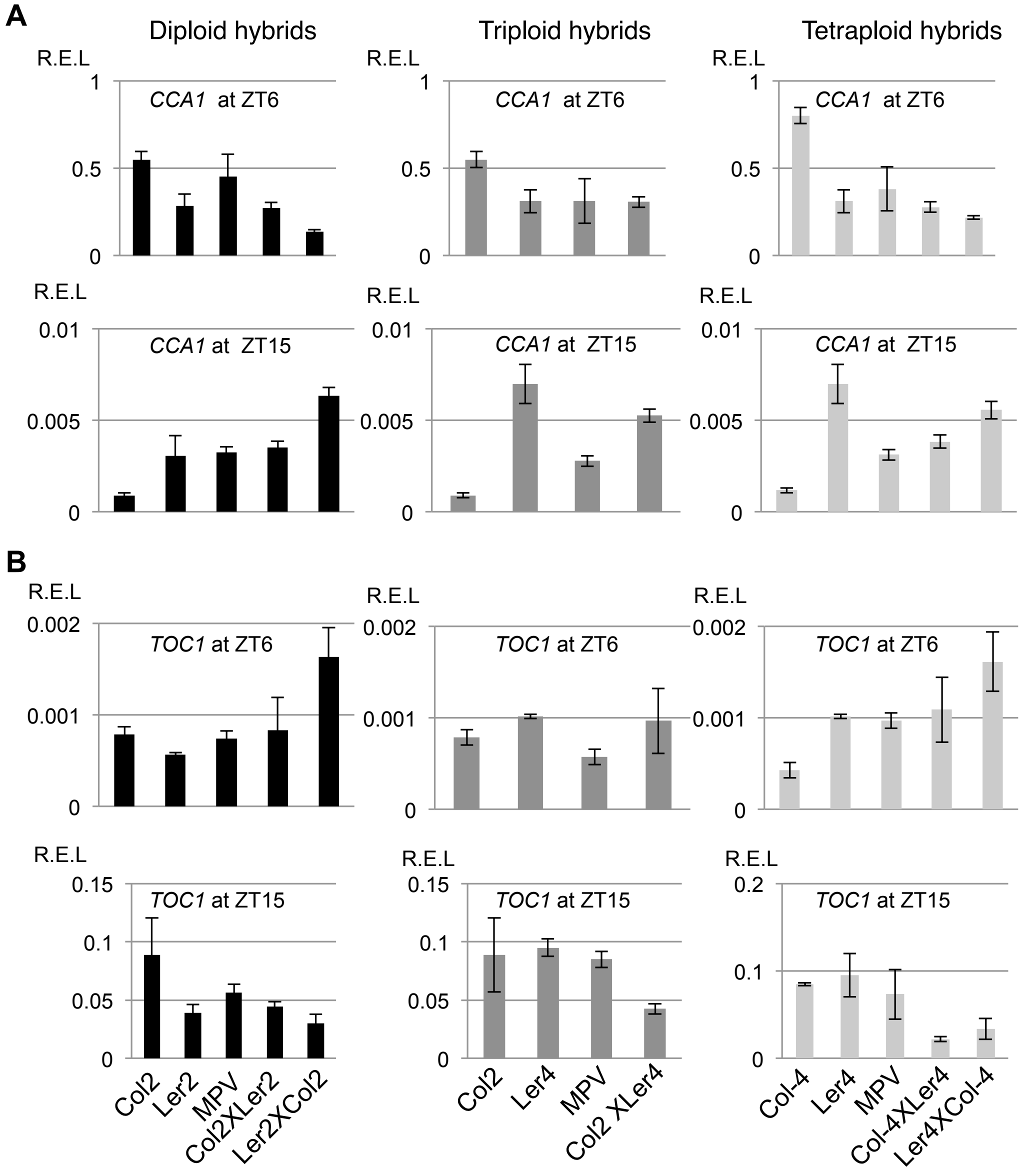

Figure S3 Expression of circadian clock genes *CCA1* and *TOC1* in ColXL*er* ploidy hybrids and their parents at ZT6 and ZT15. Quantitative RT-PCR analysis of (**A**) *CCA1* and (**B**) *TOC1* (n=3, *ACT* as an internal control). Error bars ± SD.

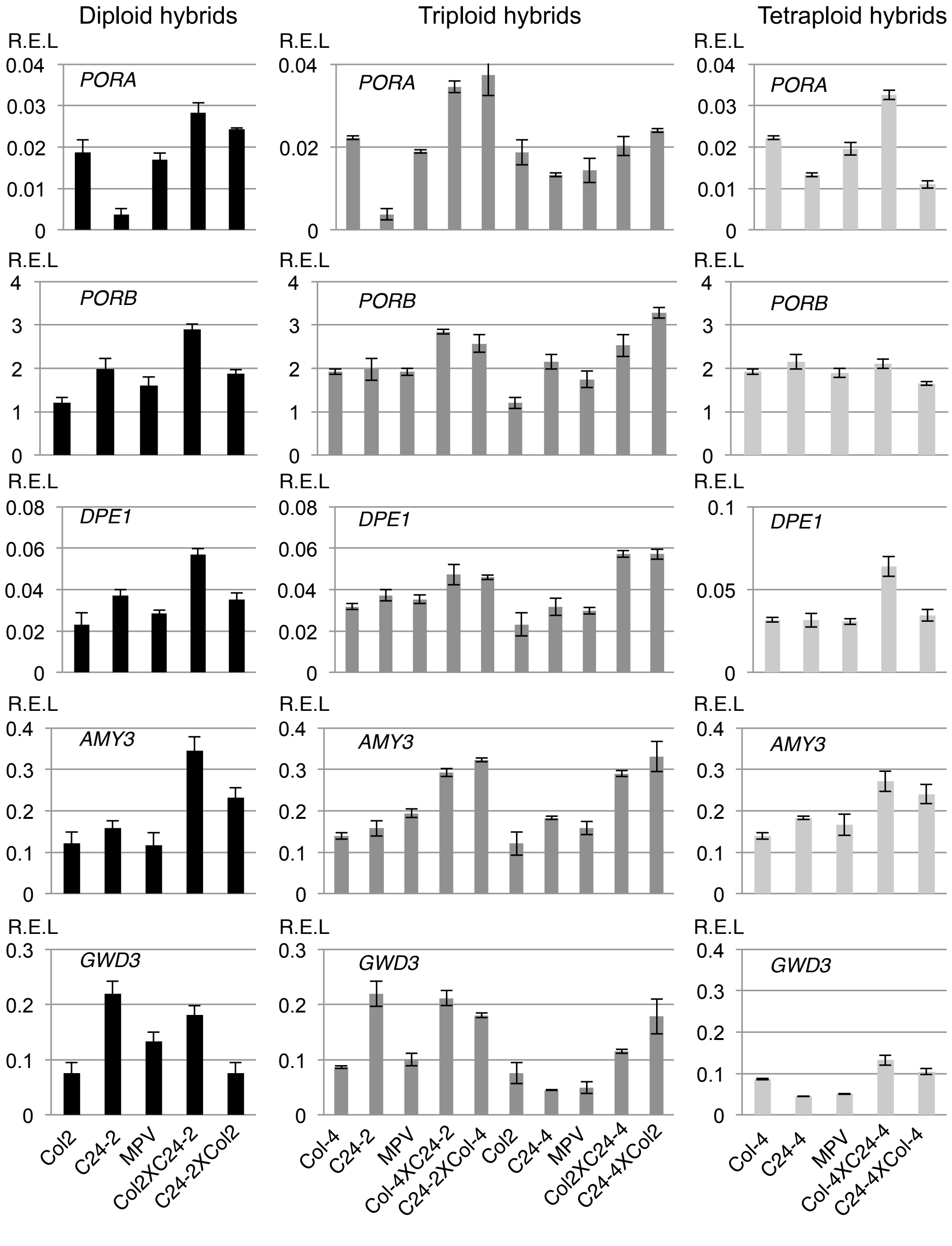

Figure S4 Expression of the genes involved in chlorophyll and starch metabolism in ColXC24 ploidy hybrids and their parents at ZT6**.** R.E. L.: relative expression levels. (n=3, *ACT* as an internal control). Error bars ± SD.

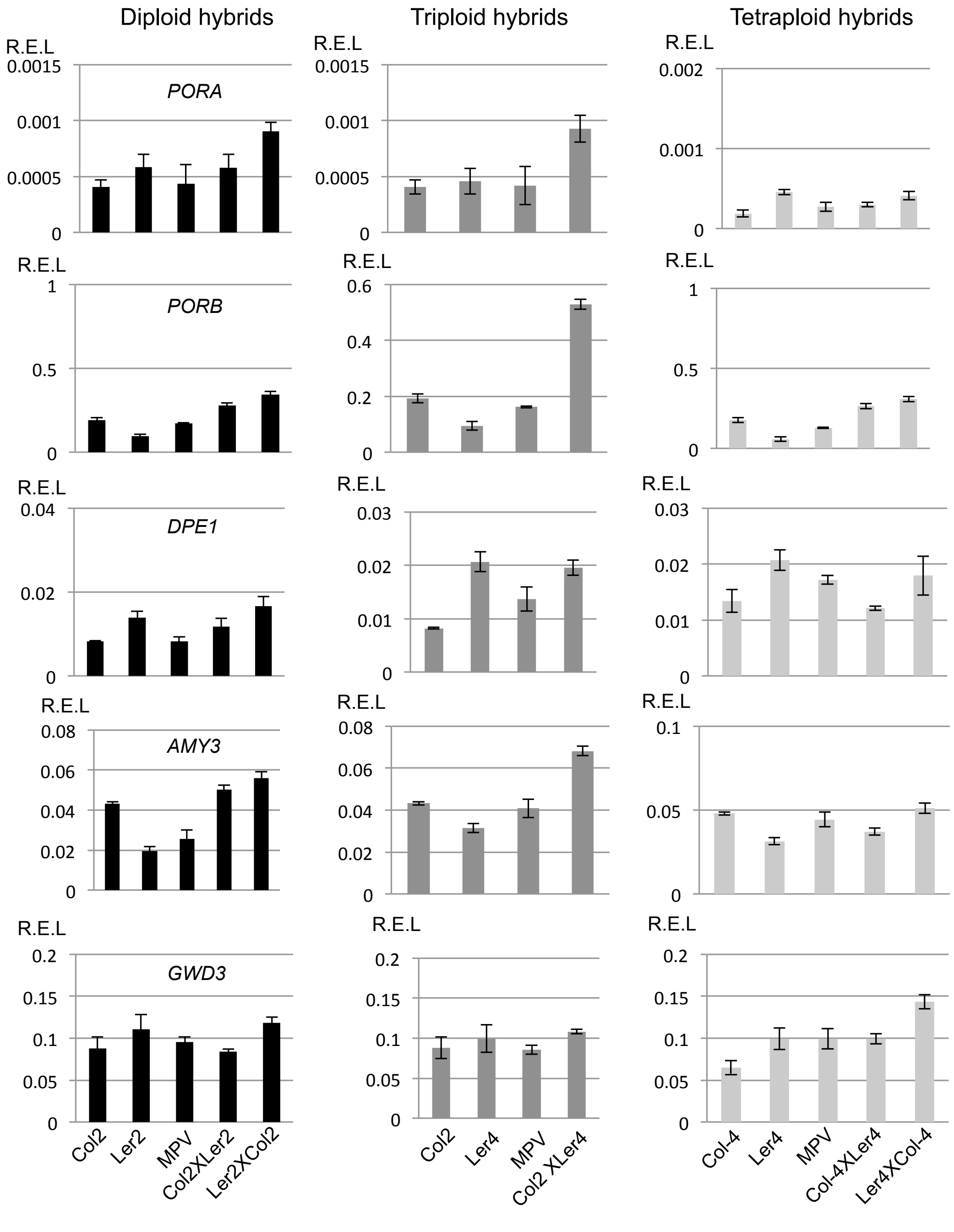

Figure S5 Expression of genes involved in chlorophyll and starch metabolism in ColXL*er* ploidy hybrids and their parents at ZT6**.** (n=3, *ACT* as an internal control). Error bars ± SD.

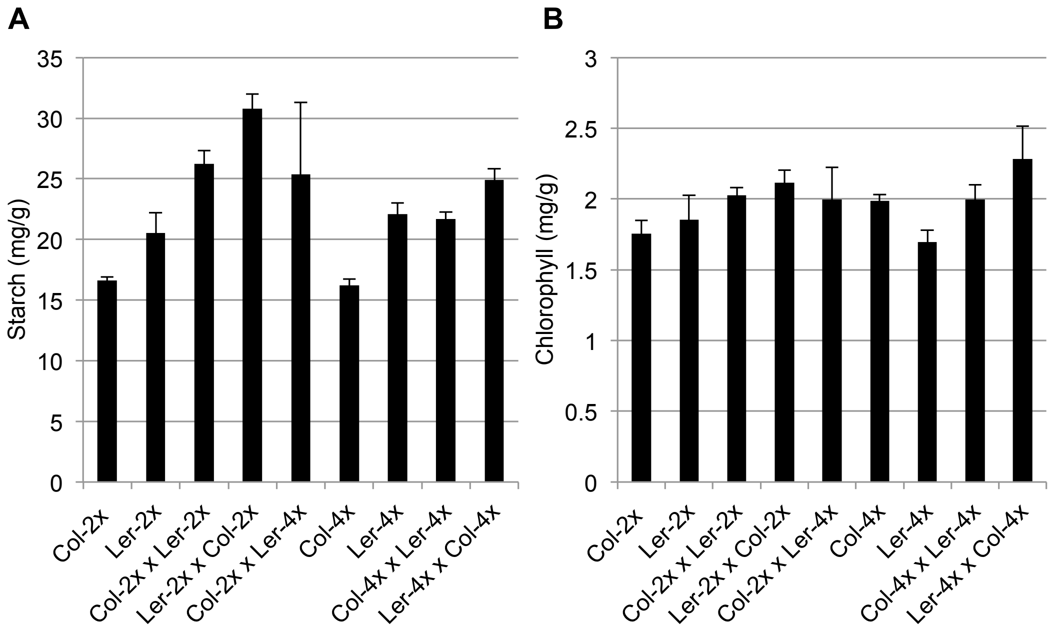

Figure S6 Starch and chlorophyll content in ColXL*er* ploidy hybrids and their parents. (**A**) Starch content (n=2) and (**B**) chlorophyll content (n=2). Error bars ± SD.

Table S1 Primer sequences of *CCA1*, *LHY*, *TOC1* and genes involved in photosynthesis and starch metabolism for quantitative RT-PCR

LOCUS	NAME	FORWARD PRIMER	REVERSE PRIMER
At2g46830	*CCA1*	5’-CCTCGTCAGACACAGACTTCCA-3’	5’-CCGCAGTAGAATCAGCTCCAATA-3’
At5g61380	*TOC1*	5’-GTTGATGGATCGGGTTTCTC-3’	5’-TCATGACCCCATGCATACAG -3’
At5g09810	*ACT*	5’-GTCTGTGACAATGGAACTGGAA-3’	5’-CTTTCTGACCCATACCAACCAT-3’
At5g54190	*PORA*	5’-GTGGTTGTCACGGGAGCTTC-3’	5’-TGCCTTTGCCGTTGCTAAAC-3’
At4g27440	*PORB*	5’-GTGGACGGCAAGAAAACGTT-3’	5’-GGCTCCAGTGACCACCACAT-3’
At1g69830	*AMY3*	5’-CTTCAAGTAGCTCGCCCGTT-3’	5’-TGGGTTTACTCACTTGGGCAG-3’
At5g64860	*DPE1*	5’-GTTCCGGATCCAGAGAGCAG-3’	5’-CGTCGGGTGTAGCAAAACG-3’
At5g26570	*GWD3*	5’-TTCGCCGGACTTATCATTCG-3’	5’-TCCGGATCAGCTGGACTCAC-3’
