# Supplementary material for: Ploidy and Hybridity Effects on Growth Vigor and Gene Expression in Arabidopsis thaliana Hybrids and Their Parents
Source: G3 (Bethesda). 2012 Apr 1;2(4):505–13. doi: 10.1534/g3.112.002162 (PMC3337479; doi:10.1534/g3.112.002162)
Supplement: Supporting Information [file supp_2.4.505_FigureS1.pdf]

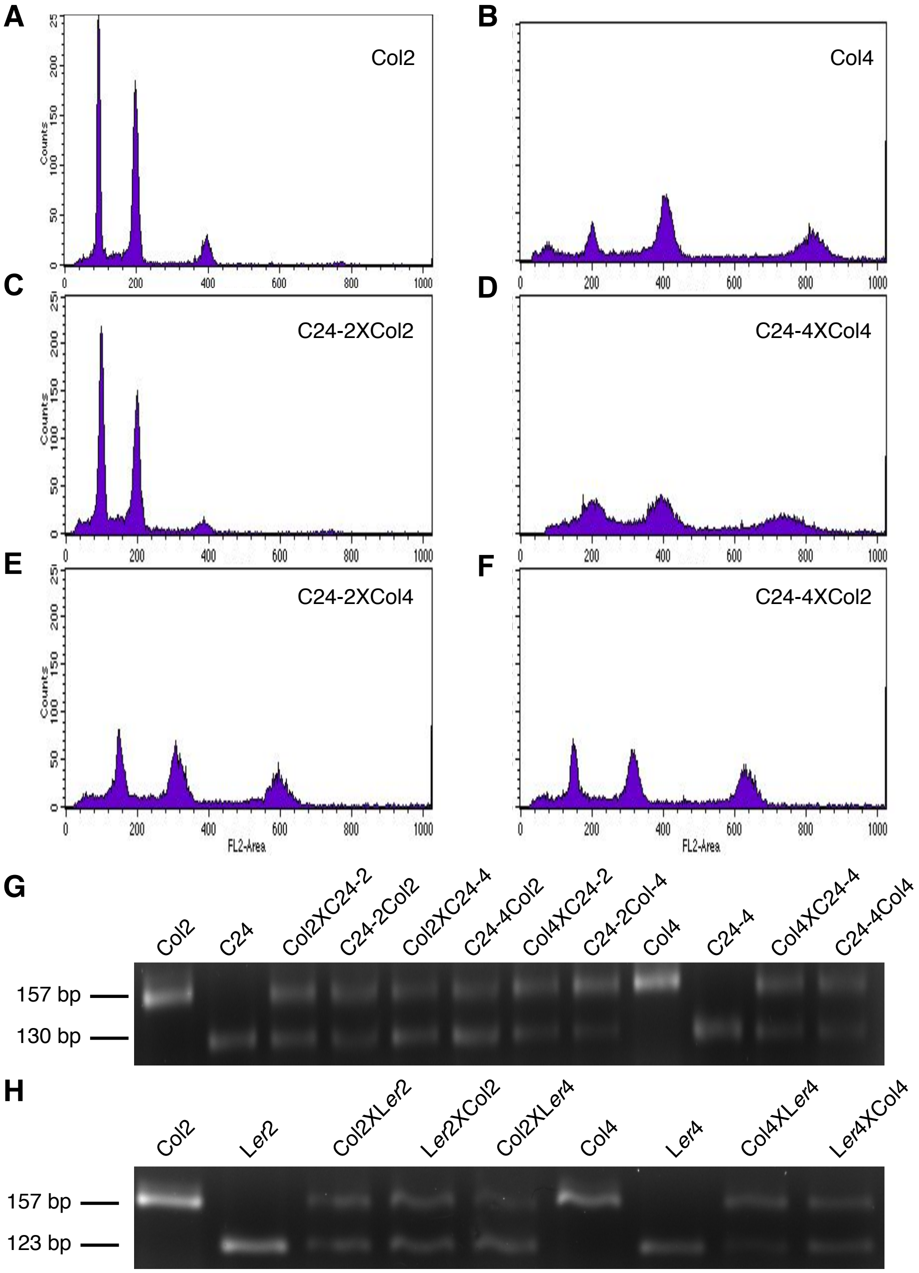

Figure S1 Validation of genotype and ploidy in ColxC24 and ColXL*er* hybrids and parents**.** (**A-F**) Flow cytometry analysis of nuclei from leaves of hybrids and parents. Filtered nuclei were stained with propidium iodide and analyzed using flow cytometry (X-axis = fluorescence intensity, Y axis = nuclei counts). (**G**) Genotyping in ColXC24 hybrids and parents using genomic DNA PCR. (**H**) Genotyping in ColXL*er* hybrids and parents using genomic DNA PCR. PCR length polymorphisms between the two different ecotypes were resolved on a 4% agarose gel to distinguish the two different genomes present in the hybrids.
