# Supplementary material for: Ploidy and Hybridity Effects on Growth Vigor and Gene Expression in Arabidopsis thaliana Hybrids and Their Parents
Source: G3 (Bethesda). 2012 Apr 1;2(4):505–13. doi: 10.1534/g3.112.002162 (PMC3337479; doi:10.1534/g3.112.002162)
Supplement: Supporting Information [file supp_2.4.505_FigureS2.pdf]

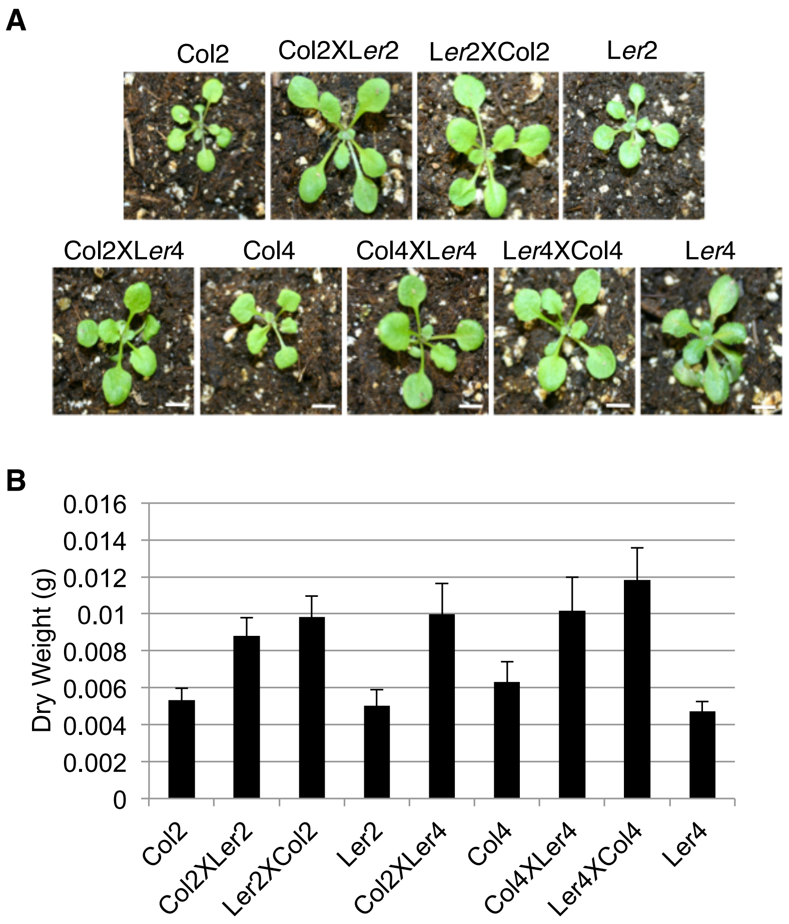

Figure S2 (**A**) Morphological vigor in ColXL*er* ploidy hybrids and their parents (bars 1 cm). (**B**) Aerial biomass in ColXL*er* ploidy hybrids and their parents. Error bars ± SD.
