# Supplementary material for: Ploidy and Hybridity Effects on Growth Vigor and Gene Expression in Arabidopsis thaliana Hybrids and Their Parents
Source: G3 (Bethesda). 2012 Apr 1;2(4):505–13. doi: 10.1534/g3.112.002162 (PMC3337479; doi:10.1534/g3.112.002162)
Supplement: Supporting Information [file supp_2.4.505_FigureS3.pdf]

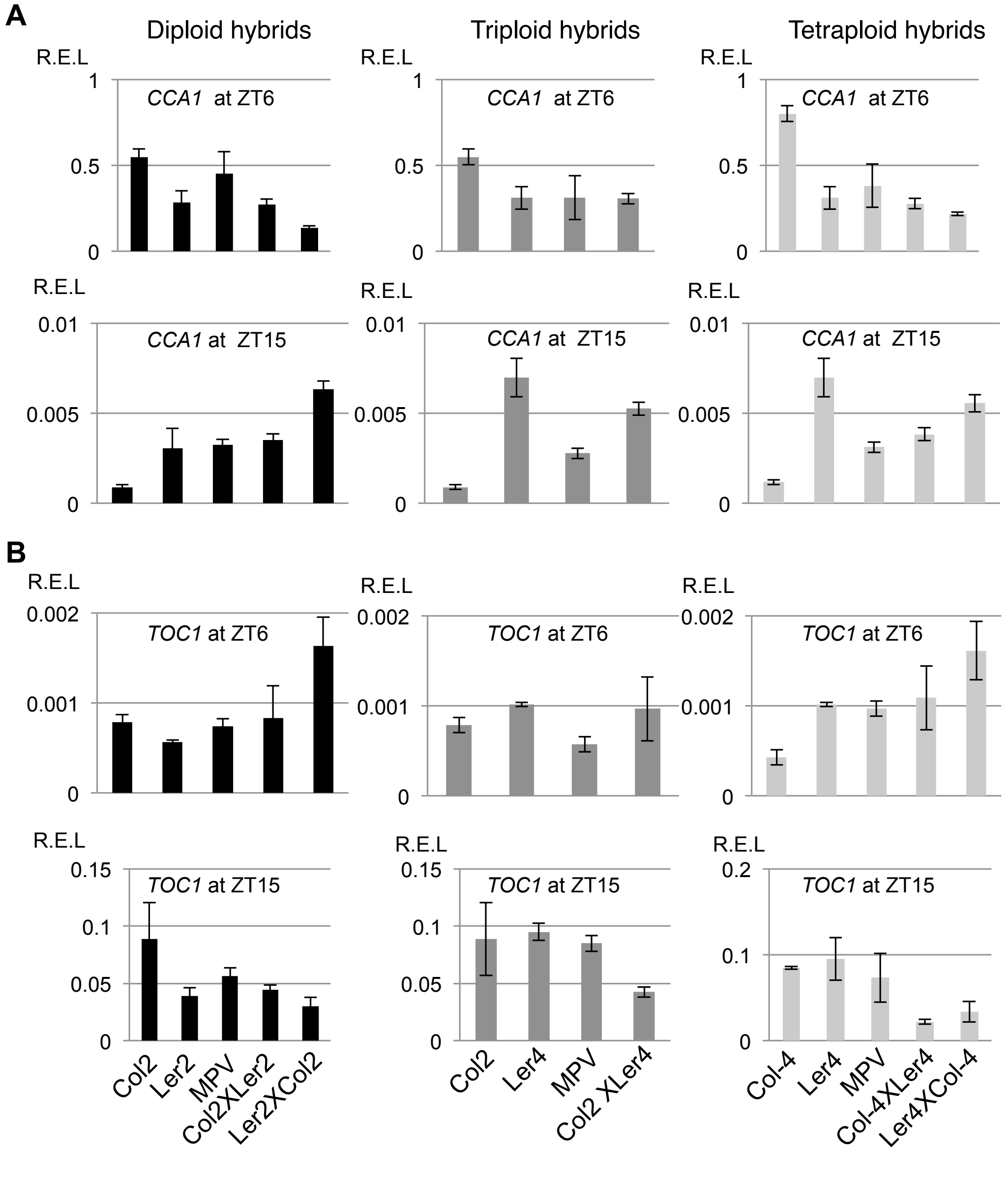

Figure S3 Expression of circadian clock genes *CCA1* and *TOC1* in ColX*Ler* ploidy hybrids and their parents at ZT6 and ZT15. Quantitative RT-PCR analysis of (**A**) *CCA1* and (**B**) *TOC1* (n=3, *ACT* as an internal control). Error bars ± SD.
