# Supplementary material for: Ploidy and Hybridity Effects on Growth Vigor and Gene Expression in Arabidopsis thaliana Hybrids and Their Parents
Source: G3 (Bethesda). 2012 Apr 1;2(4):505–13. doi: 10.1534/g3.112.002162 (PMC3337479; doi:10.1534/g3.112.002162)
Supplement: Supporting Information [file supp_2.4.505_FigureS6.pdf]

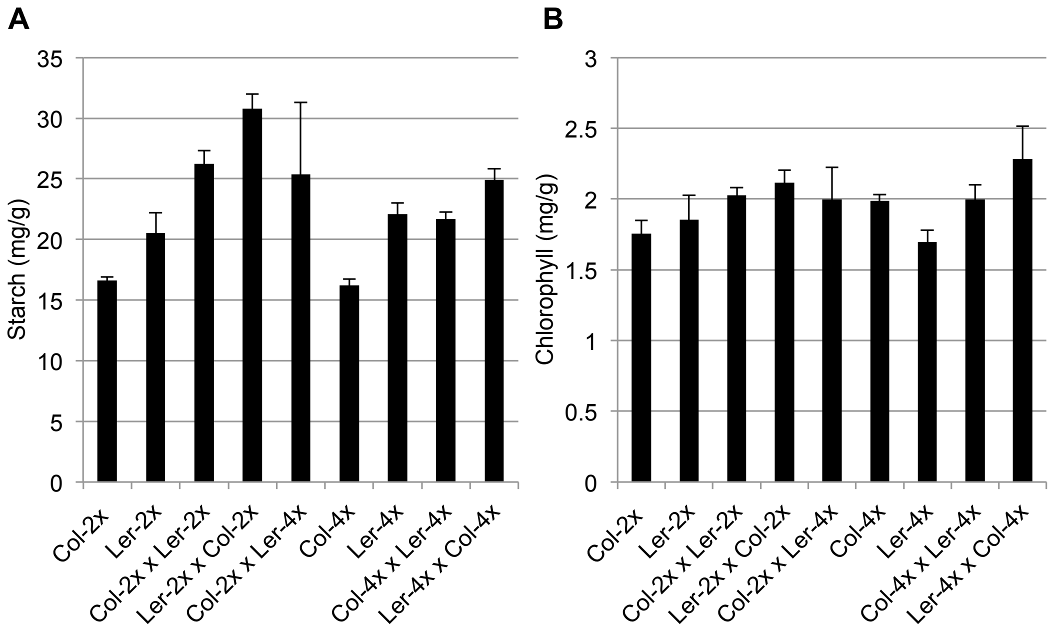

Figure S6 Starch and chlorophyll content in ColXL*er* ploidy hybrids and their parents. (**A**) Starch content (n=2) and (**B**) chlorophyll content (n=2). Error bars ± SD.
