# Supplementary material for: Ploidy and Hybridity Effects on Growth Vigor and Gene Expression in Arabidopsis thaliana Hybrids and Their Parents
Source: G3 (Bethesda). 2012 Apr 1;2(4):505–13. doi: 10.1534/g3.112.002162 (PMC3337479; doi:10.1534/g3.112.002162)
Supplement: Supporting Information [file supp_2.4.505_TableS1.pdf]

Table S1 Primer sequences of *CCA1*, *LHY*, *TOC1* and genes involved in photosynthesis and starch metabolism for quantitative RT-PCR

LOCUS	NAME	FORWARD PRIMER	REVERSE PRIMER
At2g46830	*CCA1*	5’-CCTCGTCAGACACAGACTTCCA-3’	5’-CCGCAGTAGAATCAGCTCCAATA-3’
At5g61380	*TOC1*	5’-GTTGATGGATCGGGTTTCTC-3’	5’-TCATGACCCCATGCATACAG -3’
At5g09810	*ACT*	5’-GTCTGTGACAATGGAACTGGAA-3’	5’-CTTTCTGACCCATACCAACCAT-3’
At5g54190	*PORA*	5’-GTGGTTGTCACGGGAGCTTC-3’	5’-TGCCTTTGCCGTTGCTAAAC-3’
At4g27440	*PORB*	5’-GTGGACGGCAAGAAAACGTT-3’	5’-GGCTCCAGTGACCACCACAT-3’
At1g69830	*AMY3*	5’-CTTCAAGTAGCTCGCCCGTT-3’	5’-TGGGTTTACTCACTTGGGCAG-3’
At5g64860	*DPE1*	5’-GTTCCGGATCCAGAGAGCAG-3’	5’-CGTCGGGTGTAGCAAAACG-3’
At5g26570	*GWD3*	5’-TTCGCCGGACTTATCATTCG-3’	5’-TCCGGATCAGCTGGACTCAC-3’
